# Hitchhikers on an Invader: The Parasitic Leech *Myzobdella lugubris* and the Epibiotic Barnacle *Amphibalanus improvisus* on the Atlantic Blue Crab *Callinectes sapidus* in Southwestern Europe

**DOI:** 10.1002/ece3.73410

**Published:** 2026-04-06

**Authors:** Gustavo F. de Carvalho‐Souza, Enrique González‐Ortegón, Jose A. Cuesta

**Affiliations:** ^1^ Instituto de Ciencias Marinas de Andalucía (ICMAN‐CSIC) Campus Universitario Río San Pedro Cádiz Spain; ^2^ Unidad de Investigación Asociada “Crecimiento Azul” Consejo Superior de Investigaciones Científicas (CSIC)—Instituto Andaluz de Investigación y Formación Agraria, Pesquera, Alimentaria y de la Producción Ecológica (IFAPA) Cádiz Spain

**Keywords:** biological invasions, diseases, first record, Gulf of Cadiz, non‐native species, symbiosis

## Abstract

This study reports for the first time the co‐invasion of two Western Atlantic native species, the parasitic leech *Myzobdella lugubris* and the commensal barnacle *Amphibalanus improvisus* on the invasive Atlantic blue crab *Callinectes sapidus* in southwestern Europe. Two individuals of *M. lugubris* parasitizing two male crabs were collected from the Guadalquivir River estuary (SW Spain) in August 2024 and September 2025, while *A. improvisus* was found on the carapace of an ovigerous female in April 2025. This is the first worldwide record of *M. lugubris* parasitizing *C. sapidus* in the host's non‐native range and the third European record for the leech, the first two having been documented on fish and turtles. Also, it is the first documentation of both associations with a crustacean host in European waters. Molecular analyses confirmed the identity of both non‐native species, with the *M. lugubris* COI sequence showing the highest similarity (99.85%) to a non‐native Hawaiian specimen, and 100% similarity for *A. improvisus* . Given the blood‐feeding nature of *M. lugubris* and its role as a recognized pathogen vector, the establishment of this parasitic association poses an unquantified but substantial risk for the translocation and emergence of novel infectious diseases in native European fish and crustacean populations. Furthermore, this finding highlights the need for comprehensive health surveillance of commercially exploited *C. sapidus* populations across the non‐native range.

## Graphical abstract

This study documents the first worldwide record of the parasitic leech *Myzobdella lugubris* and the commensal barnacle *Amphibalanus improvisus* associated with invasive Atlantic blue crabs ( *Callinectes sapidus* ) in southwestern Europe. Molecular analyses confirmed the identity of these Western Atlantic native species, highlighting a complex multi‐level co‐invasion in the Guadalquivir River estuary. Given the role of *M. lugubris* as a pathogen vector, this finding poses a significant risk for disease emergence in native European aquatic populations and highlights the need for comprehensive health surveillance.

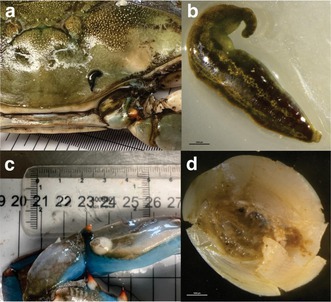

## 1 Introduction

The proliferation of non‐native species (NNS) in coastal and estuarine environments has risen sharply in the recent decades, driven predominantly by human activities such as the expansion of shipping activity and aquaculture commerce (Carlton et al. 2011; Seebens et al. 2013; Katsanevakis et al. 2018; Bray et al. 2024). These successful introductions frequently yield major disturbances of native ecosystems and provoke significant economic costs (de Carvalho‐Souza et al. 2024; Turbelin et al. 2024). Biological invasions are widely recognized as a major driver of global environmental change and a serious threat to biodiversity (Seebens et al. 2013; IPBES 2023; Bray et al. 2024).

An understanding of the mechanisms underlying the transport and dispersal of NNS is therefore crucial to predicting invasion pathways and mitigating their impacts. In marine and estuarine systems, the introduction of NNS often occurs via “hitchhiking”, defined as the unintentional transport of smaller organisms attached to, or associated with, larger hosts or substrates (Cuesta et al. 2016; Patoka et al. 2020). Such associations can involve commensal, parasitic, or epibiotic relationships that facilitate co‐invasions, where multiple NNS are introduced simultaneously through shared vectors (Daniels and Sawyer 1975; Alves‐Júnior et al. 2024).

The Atlantic blue crab *Callinectes sapidus* Rathbun, 1896, native to the western Atlantic, is a well‐known example of a successful marine invader in African and European waters (Mancinelli et al. 2017; Mabrouki et al. 2025). Since its first records in the Mediterranean and adjacent regions in the mid‐20th century (e.g., 1947 in Greece and 1949 in Italy; Mancinelli et al. 2017), *C. sapidus* has rapidly expanded its range and established stable populations in several coastal systems, from the Black Sea to the Iberian Peninsula and African waters, including insular ecosystems (Öztürk et al. 2020; González‐Ortegón et al. 2022; Mabrouki et al. 2025; UNEP/MAP‐SPA/RAC 2025). Its ecological adaptability, including trophic plasticity, tolerance to wide salinity gradients, high reproductive potential with abundant larval output, and dispersal via ballast water, among others, have contributed to its ongoing expansion (Marchessaux et al. 2022; Herrera et al. 2024; Ortega‐Jiménez et al. 2025; Rodríguez‐Ruiz et al. 2025; UNEP/MAP‐SPA/RAC 2025). However, while the crab's invasive potential has been well documented, much less is known about the co‐introduction of its associated fauna.

**FIGURE 1:**
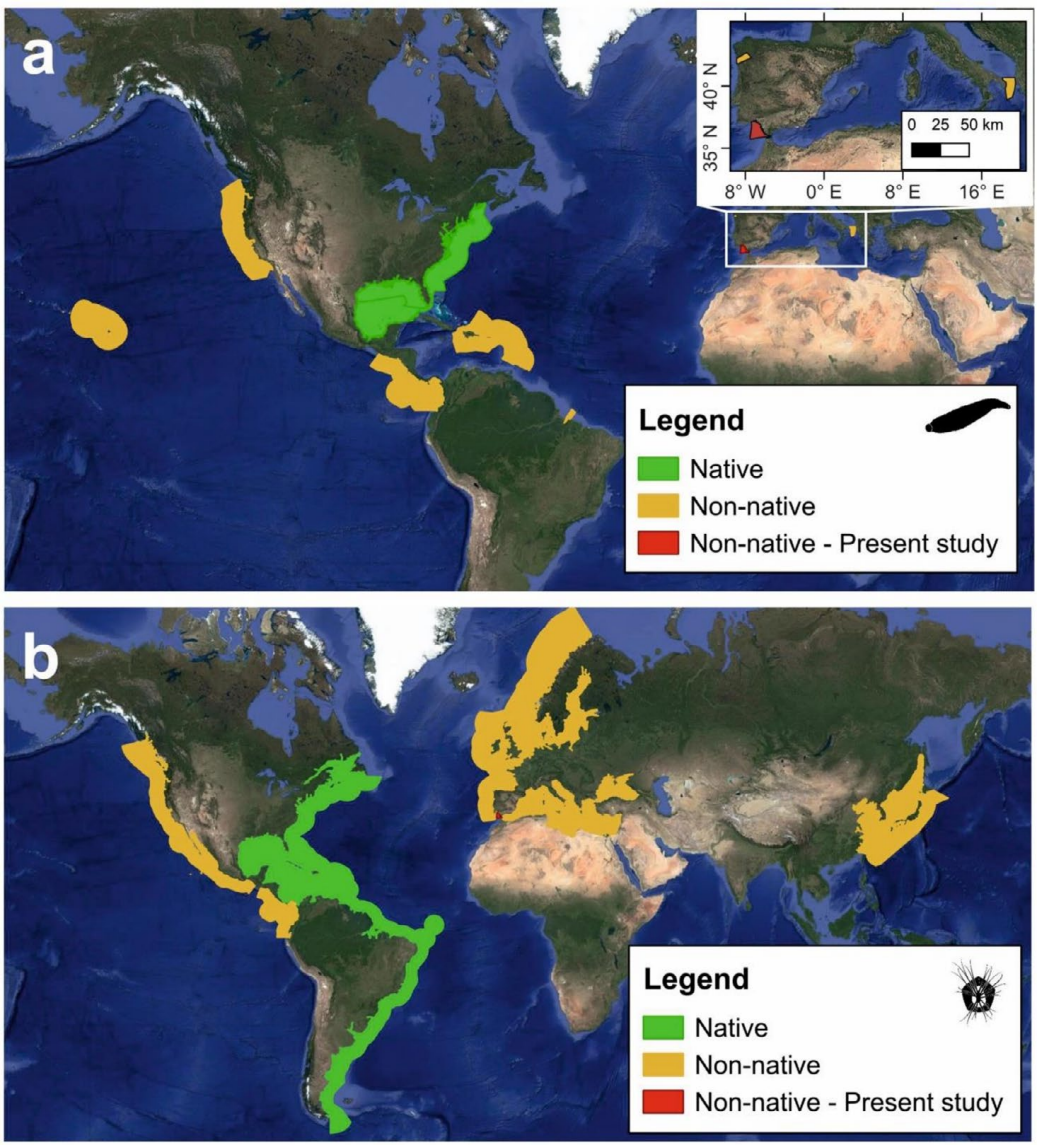
Native (green) and non‐native (yellow) distribution of *Myzobdella lugubris* (a) and *Amphibalanus improvisus* (b) (Adapted from NEMESIS database; Fofonoff et al. 2018). Red dots indicate the study location.

Epibionts and parasites of invasive hosts often represent secondary invasion risks, capable of establishing new populations in the introduced range (Shields and Overstreet 2003; Fernandez‐Leborans 2010). The parasitic leech *Myzobdella lugubris* Leidy, 1851 (Annelida: Piscicolidae), and the barnacle *Amphibalanus improvisus* (Darwin 1854) (Crustacea: Balanidae) are both native to the western Atlantic, where they are commonly associated with *C. sapidus* (Figure 1) (Daniels and Sawyer 1975; Branscomb 1976; Fofonoff et al. 2018). Yet, their occurrence in their non‐native range in association with this host has not been previously reported.

The native range of the leech *M. lugubris* is restricted to the central and eastern sectors of the Nearctic region (United States and Canada) (Fofonoff et al. 2018). This parasite exploits a wide host spectrum in estuarine habitats, including blue crabs (*Callinectes* spp.), shrimps (*Palaemon* spp.; *Penaeus* spp.), and numerous fish species (Daniels and Sawyer 1975; Sawyer 1986; Font 2003; Alves‐Júnior et al. 2024). While its distributional shifts have been documented primarily since the late 20th century, influenced by climate change and human‐mediated dispersal vectors, evidence suggests that introductions in Central and South America may have occurred earlier, potentially linked to the translocation of fish species such as the largemouth bass, *Micropterus salmoides* (Lacepède, 1802), in the 1950s and 1960s (Heidinger 1976; Santacruz et al. 2022). These shifts have led to *M. lugubris* being recorded as an invasive species (primarily on fish hosts) in the West Coast and Hawaii (USA), and Central and South America (Heidinger 1976; Williams et al. 1994; Amin and Minckley 1996; Font 2003; Troxel 2010; Oceguera‐Figueroa and Pacheco‐Chaves 2012; Fofonoff et al. 2018; Kvist et al. 2018; Santacruz et al. 2022; Alves‐Júnior et al. 2024). Although the invasive leech is known to parasitize *Callinectes* spp. in its native range (e.g., Puerto Rico and Brazil), previous European records were restricted to Italy, where it was found in brackish waters (a region where *C. sapidus* is also established), utilizing a native reptile host, the European pond turtle *Emys orbicularis hellenica* (Valenciennes, 1832) (Liuzzo et al. 2018), and more recently on fish hosts the non‐native Pumpkinseed Sunfish, *Lepomis gibbosus* (Linnaeus, 1758) in international section of the Minho River (NW‐Iberian Peninsula) (Lages et al. 2021).

The barnacle, *A. improvisus* (Darwin 1854), also considered native to the western Atlantic, is one of the most successful and widespread euryhaline biofoulers globally (Wrange et al. 2016; Fofonoff et al. 2018; Meng et al. 2024). This species was previously known by Darwin (1854) from both Atlantic coasts and the Pacific coast of tropical South America. Records from Pacific sites, such as Guayaquil, Ecuador, are thought to represent populations introduced from the Atlantic in early periods via Spanish shipping (Carlton et al. 2011). This barnacle is recognized as an ecological generalist, colonizing a broad range of hard substrates, including artificial surfaces and biological hosts such as crustaceans (Carlton et al. 2011; Naser et al. 2015). Due to its capacity for rapid settlement and tolerance to extreme environmental fluctuation, it has become an established NNS across major European water bodies, including the Mediterranean, Black, and Caspian Seas (Leppäkoski 1999; Aladin et al. 2002; Carlton et al. 2011; Naser et al. 2015). While its primary invasion vector is widely considered to be shipping (hull fouling and ballast water), its presence on a mobile, large‐bodied invasive host like *C. sapidus* can represent a highly effective secondary mechanism for local dispersal and establishment in newly colonized estuarine environments.

In this study, we document for the first time the presence of *M. lugubris* and *A. improvisus* on *C. sapidus* in southwestern Europe, within the Guadalquivir River estuary (SW Spain). We further discuss the implications of these findings for understanding co‐invasion dynamics and the potential role of such associations in the translocation of pathogens across biogeographic boundaries.

## 2 Material and Methods

**FIGURE 2:**
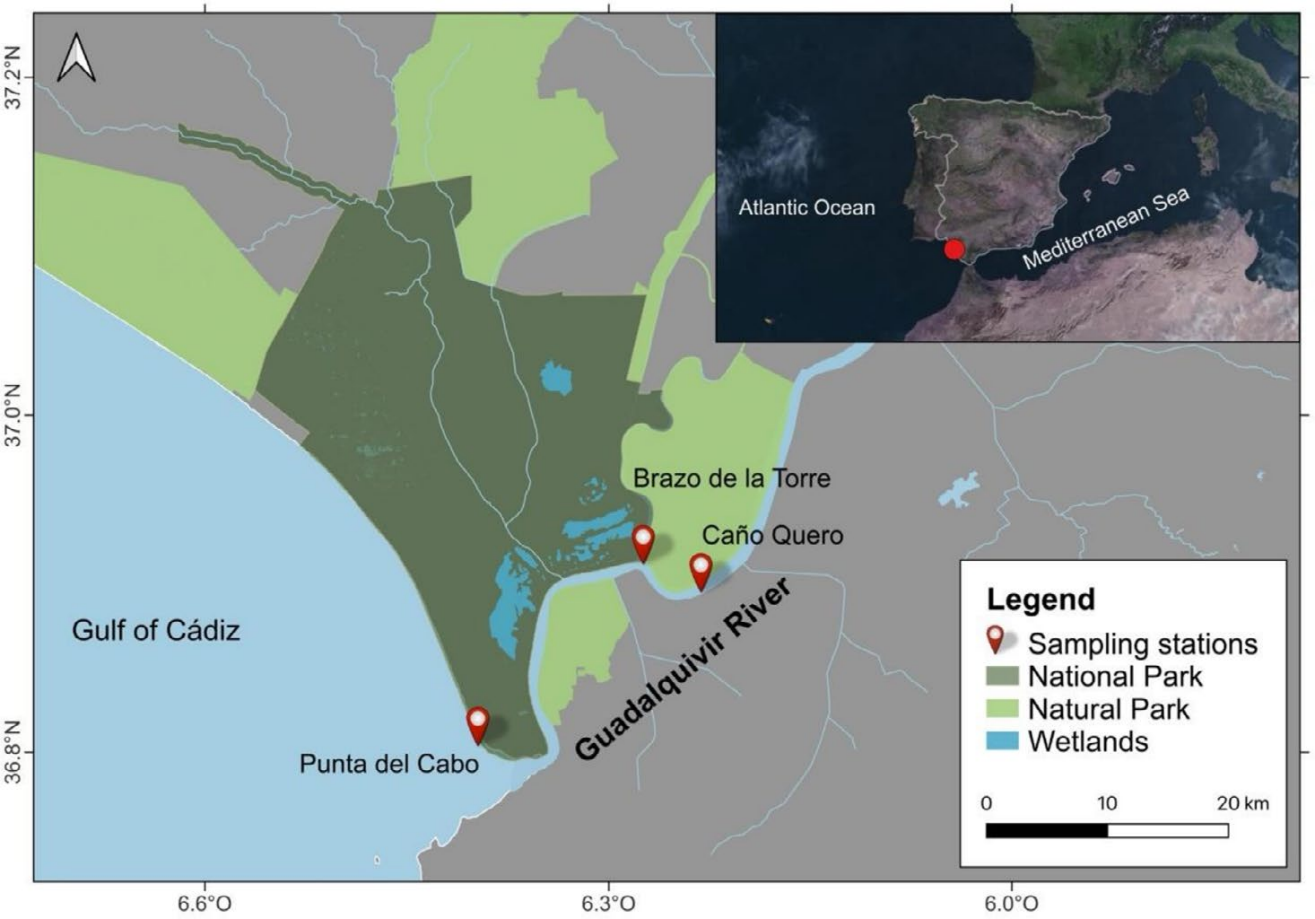
Map of the Guadalquivir River estuary (SW Spain) and locations of the three sampling stations: Punta del Cabo, Brazo de la Torre, and Caño Quero.

Specimens of the Atlantic blue crab *C. sapidus* were collected from local fisheries landings in the Guadalquivir River estuary, SW Spain, as part of a monitoring program (de Carvalho‐Souza et al. 2024) spanning from June 2024 to December 2025. Sampling was conducted across a longitudinal gradient of the estuary, encompassing stations from the mouth (Punta del Cabo) up to approximately 20 km to 25 km upstream (e.g., Brazo de la Torre and Caño Quero) (Figure 2). Sampling gear consisted of a cylindrical baited trap net (4.5 m in length; 50 cm in diameter; 6 cm mesh size), deployed at an approximate depth of 1 m, baited with a chicken carcass, and retrieved after 24 h.

The collection yielded three host individuals associated with non‐native fauna: two male crabs, collected in August 2024 and September 2025, each parasitized by a single leech specimen, and one ovigerous female crab, collected in April 2025, bearing an epibiotic barnacle specimen attached to its carapace. Meristic and morphometric measurements were taken for both the host and the associated fauna. All epibiont specimens were preserved and deposited in the reference collection of the Spanish Institute of Oceanography (CRUST‐IEO, Cádiz, Spain; Muñoz and Serrano 2025) under the accession numbers CRUST_PARAS/4292 and CRUST_PARAS/4293. To assess the infestation rate, prevalence was calculated for the entire study period. Given the expected low prevalence, the exact binomial probability estimation (Clopper‐Pearson method) with a 95% confidence interval (CI) was calculated for both the overall background population and per specific site, using the exactci package in R version 4.4.3 (Fay 2010; R Core Team 2025).

Species were identified through integrated morphological and molecular approaches. The specimens were previously identified using morphological descriptions from Moore (1946), Henry and McLaughlin (1975), and Saglam et al. (2018). For taxonomic confirmation, DNA was extracted from internal tissue samples of one leech and the barnacle specimen using the Qiagen DNeasy Blood & Tissue Kit following the manufacturer's protocol. The target mitochondrial genes, 16S rRNA and COI, were amplified for the two specimens, the leech and the barnacle, via Polymerase Chain Reaction (PCR). Cycling conditions of PCR were: 15 min at 95°C, 40 cycles of 30s at 95°C, 30s at 45°C (16S)/35 s at 43/51°C (COI), and 30s at 72°C, and finally 5 min at 72°C. Primers 1472 (5′‐AGA TAG AAA CCA ACC TGG‐3′) (Crandall and Fitzpatrick 1996) and 16 L2 (5´‐TGC CTG TTT ATC AAA AAC AT‐3′) (Schubart et al. 2002) were used to amplify a maximum of 532 bp of 16S, while primers COH6 (5′‐TAD ACT TCD GGR TGD CCA AAR AAY CA‐3′) and COL6b (5′‐ACA AAT CAT AAA GAT ATY GG‐3′) (Schubart and Huber 2006), and LCO1490‐JJ (5′‐CHA CWA AYC ATA AAG ATA TYG G‐3′) and HCO2198‐JJ (5′‐AWA CTT CVG GRT GVC CAA ARA ATC A‐3′) (Astrin and Stüben 2008) allowed amplification of a maximum of 658 bp of COI. PCR products were purified, bidirectionally sequenced by Stab‐Vida Laboratories, and edited using Chromas version 2.0. Final sequences were validated via BLAST searches on the NCBI website and deposited in the GenBank database under the accession numbers PX611836 and PX611837 (16S) and PX611839‐PX611840 (COI) for *M. lugubris* and *A. improvisus* , respectively.

## 3 Results

A total of three Atlantic blue crab *C. sapidus* specimens, collected within the Guadalquivir River estuary, were found to host non‐native associated fauna (Table 1). The records confirmed the presence of the parasitic leech *M. lugubris* and the epibiotic barnacle *A. improvisus* . During the monitoring period from June 2024 to December 2025, a total of 3224 blue crabs were observed across the three estuarine stations. The background crab population exhibited an average carapace width of 14.15 ± 2.06 cm and an average weight of 225.13 ± 99.46 g. Sampling effort varied slightly by location, with 1601 crabs collected at Punta del Cabo, 887 at Brazo de la Torre, and 736 at Caño Quero. Overall, the prevalence for both associated species was extremely low. Only two individuals were parasitized by *M. lugubris* , yielding an overall prevalence of 0.06% (Exact Binomial 95% CI: 0.007%–0.22%). Site‐specific prevalence for the leech was 0.11% (95% CI: 0.003%–0.63%) at Brazo de la Torre and 0.14% (95% CI: 0.003%–0.75%) at Caño Quero. Similarly, *A. improvisus* was detected on a single crab, representing an overall prevalence of 0.03% (95% CI: 0.0008%–0.17%), with a site‐specific prevalence of 0.06% (95% CI: 0.002%–0.35%) at Punta del Cabo.

**TABLE 1:** Morphometric data and collection details for the Atlantic blue crab ( *Callinectes sapidus* ) hosts and their associated non‐native fauna collected in the Guadalquivir River estuary, SW Spain. Host measurements include Carapace Width including lateral spines (CW), Carapace Length (CL), and Weight. Associated fauna Size indicates the length of the leech ( *Myzobdella lugubris* ) or the lateral and rostro‐carinal diameter of the barnacle (*Amphibalanus improvisus*).

Date	Location	Host				Associated fauna	
		Sex	CW (cm)	CL (cm)	Weight (g)	Species	Size (mm)
22/08/2024	Caño Quero	Male	14.1	6.3	202.5	*M. lugubris*	12
12/09/2025	Brazo de la Torre	Male	17.0	7.4	335.9	*M. lugubris*	9.5
22/04/2025	Punta del Cabo	Ovigerous Female	18.5	7.4	351	*A. improvisus*	6.7/7.0

**FIGURE 3:**
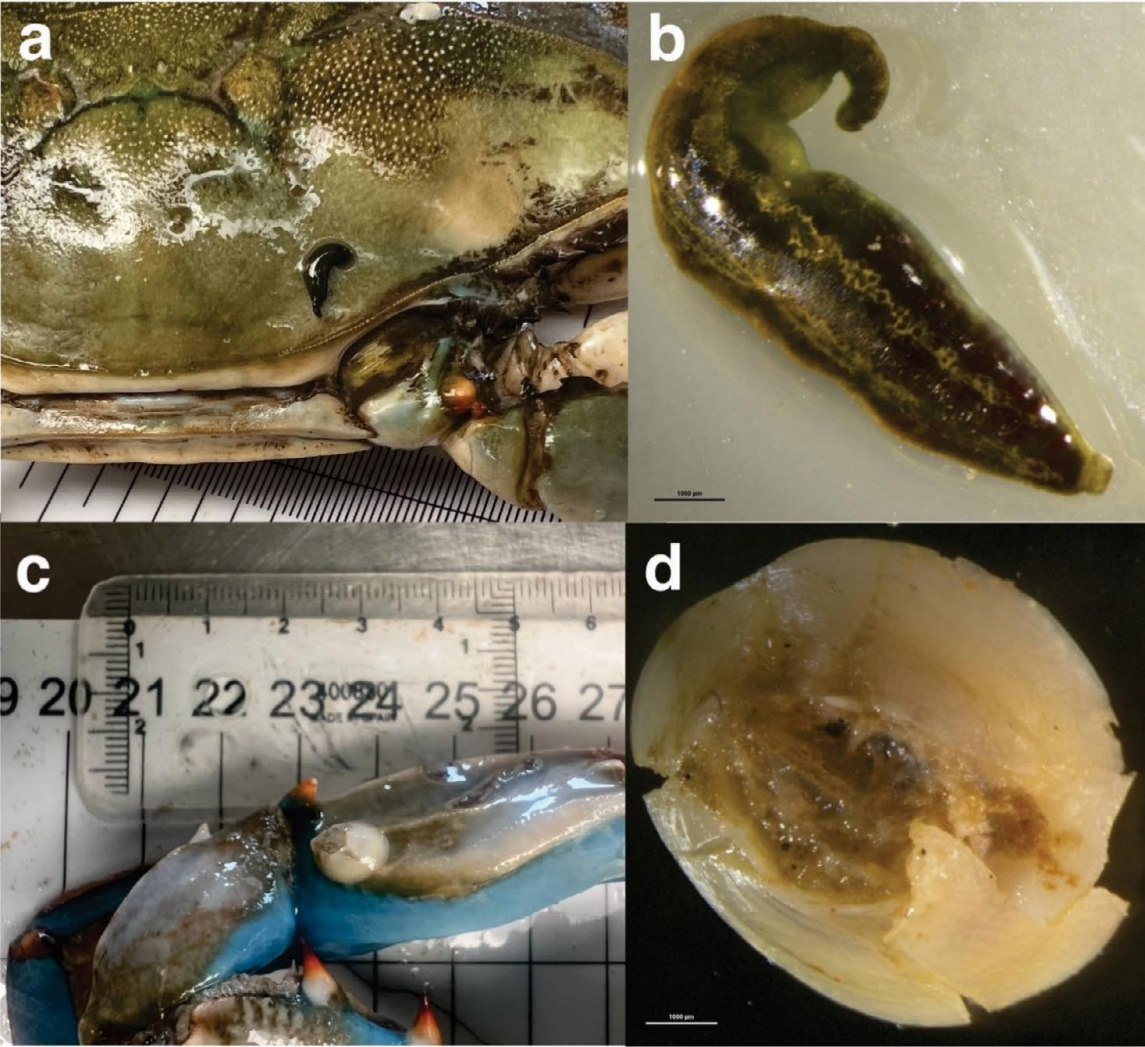
Non‐native species associated with *Callinectes sapidus* in the Guadalquivir River estuary, SW Spain. (a) The parasitic leech, *Myzobdella lugubris* , observed in situ on the carapace of the Atlantic blue crab host. (b) Close‐up view of a *M. lugubris* specimen. (c) Epibiotic barnacle, *Amphibalanus improvisus*, settled on the left chela of the Atlantic blue crab. (d) Detailed view of *A. improvisus* specimen. Scale bars, (b,c = 1 mm).

Two male *C. sapidus* individuals were parasitized by a single *M. lugubris* specimen each (Figure 3a,b). The first parasitized male, collected on August 22, 2024, at Caño Quero station (36°89′50.16″N 6°23′15.56″W), hosted a leech measuring 12.0 mm in length (Table 1). The leech was specifically attached to the crab's carapace in the region of the right anterolateral teeth. The second male host, collected on September 12, 2025, at Brazo de la Torre station (36°91′16.88″N 6°27′44.23″W), was larger and hosted a leech measuring 9.5 mm in length (Table 1). This second specimen was found attached to the carapace, close to the base of the right fifth ambulatory leg. Both individuals did not have cocoons (the egg cases of the leech) attached to the carapace. The barnacle *A. improvisus* was found on the propodus of the left chela of one large ovigerous female *C. sapidus* (Figure 3c,d), collected on April 22, 2025, at Punta del Cabo (36°80′37.40″N 6°39′72.97″W; Table 1). This ovigerous female carried brown eggs, indicative of a late developmental stage prior to hatching. The *A. improvisus* specimen found on the chela appendage exhibited a rostro‐carinal diameter of 7.0 mm and lateral of 6.7 mm.

Molecular confirmation was obtained for both species. BLAST analysis of the *M. lugubris* COI sequence showed the closest similarity (99.85%) to a sequence from Hawaii (GenBank: DQ414325), differing by only one mutation, and 99.67% similarity with a sequence from Italy (GenBank: MG820612), with a divergence of two mutations. The sequence also showed 99.03% similarity (six mutations) to one from Nicaragua (GenBank: PP434472). However, sequences from the native range (USA and Mexico) showed equal or superior divergence (2 to 16 mutations). Notably, this study provides the first GenBank deposit of a 507 bp 16S rRNA sequence for *M. lugubris* . For *A. improvisus* , the COI sequence showed the highest similarity (99.54%) with sequences from the USA (GenBank: MK308210) and the Arctic Ocean (GenBank: MG311522), with a divergence of three mutations in 658 bp in both cases, and 99.39% with European records (Germany, Genbank: KT209057, UK, GenBank: OZ188183 and Finland, GenBank: OQ644552). The 16S rRNA sequence for *A. improvisus* demonstrated 100% similarity with two mitogenomes sequences of *A. improvisus*, one from the UK (GenBank: OZ188183), and another without locality data (Genbank: MZ049958). The rest of Genbank 16S sequences of *A. improvisus* are eight shorter sequences (379–468 bp) with 100% similarity too, except one (Genbank: FJ862079), that differ in one mutation in 468 bp.

## 4 Discussion

The detection of the parasitic leech *M. lugubris* and the epibiotic barnacle *A. improvisus* on *C. sapidus* populations in southwestern Europe highlights a complex case of multi‐level biological invasions. Both non‐native hitchhikers, appear to have a high capacity for spread, mirroring the remarkable invasive success of their host and creating novel parasitic and commensal associations in European coastal ecosystems. Invasive hosts often act as vectors for translocated parasites and symbionts, thereby facilitating “invasional meltdown” processes in recipient ecosystems (e.g., Goedknegt et al. 2015; Ricciardi and Simberloff 2025). Recent molecular studies (e.g., Torres‐Carrera et al. 2024) have highlighted significant cryptic diversity and taxonomic complexities within the genus *Myzobdella*. Consequently, the use of molecular markers (COI and 16S) was necessary in the present study to accurately confirm the identity of the Iberian specimens as *M. lugubris* and distinguish them from closely related taxa.

This study reports two species known to associate with *C. sapidus* in its native range, *M. lugubris* and *A. improvisus* , utilizing the invasive Atlantic blue crab as a vector in the Guadalquivir River estuary. It also represents the third European record of *M. lugubris* , the second from the Iberian Peninsula, and the first documentation of its association with a crustacean host in one of its non‐native ranges, the European waters. The earlier European records, reported from Italy and NW‐Iberian Peninsula, involved the leech parasitizing the native European pond turtle ( *Emys orbicularis hellenica* ) (Liuzzo et al. 2018) and the non‐native Pumpkinseed Sunfish ( *Lepomis gibbosus* ) in the international section of the Minho River (Lages et al. 2021). Our finding on *C. sapidus* suggests a host‐switching event in the introduced range or, more likely, reinforces that the primary mode of introduction is directly linked to the crab. While *M. lugubris* is known to be a generalist parasite, the fact that its three European records involve three phylogenetically distinct hosts (Perciformes, Reptilia and Decapoda) reveals its marked ecological plasticity and potential to colonize diverse native fauna in the new environment.

The introduction of *M. lugubris* into the Guadalquivir estuary could have been likely mediated by the same vectors responsible for the arrival and ongoing expansion of its primary host, *C. sapidus* . Established pathways for *C. sapidus* dispersal include larval transport via ballast water and the translocation of adult individuals through hull fouling community or unauthorized aquaculture activities (Mancinelli et al. 2017; González‐Ortegón et al. 2022), and westward expansion from the Mediterranean across the Strait of Gibraltar, as the species is an excellent swimmer (Mancinelli et al. 2017; González‐Ortegón et al. 2022). However, the arrival of *M. lugubris* into the Guadalquivir River estuary may also be linked to freshwater pathways. In its native and non‐native ranges in America, as well as in the recently documented case in the Minho River (Iberian Peninsula), *M. lugubris* has been found parasitizing exotic centrarchids, specifically the largemouth bass ( *M. salmoides* ) and the pumpkinseed sunfish ( *L. gibbosus* ) (Lages et al. 2021; Santacruz et al. 2022). Both fish species are popular in recreational fishing and the aquarium trade and have established invasive populations in the Guadalquivir basin (Granado‐Lorencio 1991; Almeida and Grossman 2014). Therefore, the translocation of these infected exotic fish, either through intentional stocking for angling or accidental releases, constitutes a highly feasible alternative pathway for the arrival of the leech in the region. Once introduced, *M. lugubris* could have subsequently colonized the Atlantic blue crab, utilizing *C. sapidus* as a secondary host and a mobile substrate for further dispersal within the estuary.

Given that *M. lugubris* commonly parasitizes *C. sapidus* and *C. bocourti* in their native Western Atlantic range (Daniels and Sawyer 1975; Williams et al. 1994; Alves‐Júnior et al. 2024; Table 2), this relationship should not be ruled out for this co‐invasion mechanism. The crab's high mobility facilitates the hitchhiking of adult leeches or via cocoons attached to the crab's carapace, bypassing traditional dispersal barriers, even if cocoons were not observed in this study.

**TABLE 2:** Documented records of *Myzobdella lugubris* on the crab host in its non‐native range. Acronym: N—Number of individuals; Number of hosts infected (Hi) per number of hosts examined (He); USNM—Smithsonian Institution, National Museum of Natural History (https://www.si.edu/about/natural‐history‐museum); CRUST‐IEO—Reference collection of the Spanish Institute of Oceanography.

Date	Host	*N*	Site	Hi/He	Host size (cm)	Locality	Collection number	References
29/05/1992	*C. bocourti*	4–10	body	17–17	7–7.5	Santa Teresa Lagoon, Puerto Rico	USNM 155347	Williams et al. (1994)
29/05/1992	*C. sapidus*	6	body	1–1	7.9	Santa Teresa Lagoon, Puerto Rico	—	Williams et al. (1994)
12/11/1995	*C. bocourti*	1	leg	1–1	12.9	Arecibo, Puerto Rico	USNM 172128	Smithsonian Database
22/08/2024	*C. bocourti*	1–21	Carapace; cheliped	86–86	6.1–13.7	Curuçá, Pará, Brazil	—	Alves‐Júnior et al. (2024)
22/08/2024	*C. sapidus*	1	Carapace	1–1	14.1	Caño Quero, Guadalquivir River, Spain	CRUST‐IEO	Present study
12/09/2025	*C. sapidus*	1	carapace	1–1	17.0	Brazo de la Torre Guadalquivir River, Spain	CRUST‐IEO	Present study

The association records occurred during the spring and summer, when estuarine temperatures rise, with summer peaks reaching 28°C. This corresponds to the typical association period in its native range, which occurs under low salinity and relatively high‐water temperature (Daniels and Sawyer 1975; Sawyer 1986). The stations where leeches were collected, Caño Quero and Brazo de la Torre, show mean salinity values fluctuating between 5 and 15, whereas Punta del Cabo station, in the mouth of the Guadalquivir River estuary presenting salinity levels varies 17 and 27 (González‐Ortegón et al. 2023). For comparison, the sympatric leech, *M. platensis* (Cordero, 1933) exhibits higher infestation rates (prevalence > 88.0%) on *C. sapidus* in a Brazilian estuary, particularly at lower salinities and on larger individuals (maximum CW of 16.8 cm; Severino‐Rodrigues and de Almeida‐Duarte 2020). This pattern, consistent with our size observations, suggests that beyond salinity, larger individuals are favored not only because they offer greater carapace area for leech attachment and cocoon deposition, but also because they exhibit longer intermolt periods, allowing the epibiont/parasite greater duration for growth and colonization. However, based on the number of specimens observed monthly in the present monitoring (more than 3200 specimens; de Carvalho‐Souza et al. 2024), the parasitic prevalence is low. While this low density might indicate an early stage of arrival, it is also plausible that *M. lugubris* currently acts as an occasional, facultative associate of the blue crab in this newly invaded region.

While *A. improvisus* is a globally distributed and well‐established NNS in European coastal waters (Carlton et al. 2011; Naser et al. 2015), its successful colonization via epibiotic association with a large, mobile, invasive decapod host like *Callinectes* spp. has significant implications for local dispersal dynamics (Alves‐Júnior et al. 2023). In the Guadalquivir estuary, *C. sapidus* is broadly distributed across the salinity gradient and moves actively between salinity zones, which could facilitate the simultaneous transfer of its epibionts (including *A. improvisus* ) into previously unoccupied mesohaline or oligohaline zones. Although *A. improvisus* is already a widespread associate of crustaceans, this mechanism may provide an additional secondary dispersal route for the barnacle, potentially aiding its local movements alongside traditional dispersal via planktonic larvae or hull fouling (given the mobility of swimming crab) (Carlton et al. 2011). It is important to note, however, that the crustacean exoskeleton serves only as a temporary substratum for epibionts because ecdysis necessitates the periodic discarding of the carapace into the environment, whereupon the attached organisms are typically eliminated by physical forces (waves and currents), sedimentation, or predation (Alves‐Júnior et al. 2023).

The occurrence of *M. lugubris* adds an additional ecological risk to the recipient ecosystem, particularly considering the pre‐existing pathogen load associated with the invasive host. In its native range, *C. sapidus* harbors a diverse assemblage of symbionts and pathogens, including viruses, bacteria, fungi, protozoans, and helminths (Shields and Overstreet 2003). The parasitic dinoflagellate *H. perezi* , a known pathogen of decapods, is already established in invasive Atlantic‐Mediterranean populations of blue crab with high prevalences (up to 85%) (Mancinelli et al. 2017; Lamkhalkhal et al. 2024; Lattos et al. 2024; Schott et al. 2025) and has been implicated in mortality events and population declines in Greece (Lattos et al. 2024).

Beyond the direct ecological impacts posed by the establishment of *M. lugubris* and *A. improvisus* , a potential concern associated with this co‐invasion event is the theoretical capacity of *M. lugubris* to facilitate pathogen introductions or transmission. Leeches, as obligate blood‐feeding parasites, are widely recognized as competent vectors for a variety of blood‐borne microorganisms, including bacteria, protozoans, and viruses (e.g., Phillips et al. 2020). In the native range, high infestation rates of *M. lugubris* are often linked to lesions, reduced host fitness, and secondary infections in crustacean and fish. The feeding process can trigger histopathological responses and is known to facilitate the transmission of opportunistic bacterial pathogens, including *Flavobacterium* sp., *Pseudomonas* sp., *Bacillus* sp., and *Staphylococcus* sp. (Volonterio et al. 2004; Noga et al. 1990; Kikuchi and Fukatsu 2005; Goffredi et al. 2012; Pomposini et al. 2019).

Consequently, the presence of a novel, widespread parasitic vector such as *M. lugubris* in the Guadalquivir estuary could supposedly present an unquantified risk of introducing pathogens carried by the invasive *C. sapidus* or acquired from native European fauna via host‐switching. The finding of *M. lugubris* on a new mobile host in a new region highlights the need for urgent follow‐up research focused on characterizing the microbiome and pathogen load associated with both the parasite and its invasive host (Phillips et al. 2020; Lattos et al. 2024; Schott et al. 2025). Furthermore, given the increasing commercialization of *C. sapidus* across Africa and Europe (de Carvalho‐Souza et al. 2024; UNEP/MAP‐SPA/RAC 2025), the potential for these infectious agents to impact fisheries and trade cannot be ignored. A comprehensive health surveillance strategy for *C. sapidus* is necessary across its non‐native range, as monitoring this leech‐crab association is critical for assessing the potential emergence of novel infectious diseases in native fish and crustacean populations.

## Author Contributions

**Gustavo F. de Carvalho‐Souza:** conceptualization (equal), data curation (equal), formal analysis (equal), investigation (equal), methodology (equal), visualization (equal), writing – original draft (lead). **Enrique González‐Ortegón:** conceptualization (equal), funding acquisition (equal), investigation (equal), methodology (equal), resources (equal), supervision (equal), writing – review and editing (equal). **Jose A. Cuesta:** conceptualization (equal), data curation (equal), formal analysis (equal), investigation (equal), supervision (equal), writing – review and editing (equal).

## Disclosure

Data: Sequence data were deposited in GenBank and their IDs are provided in the text.

## Conflicts of Interest

The authors declare no conflicts of interest.

## Data Availability

Sequence data were deposited in GenBank and their IDs are provided in the text.
